# Quantitative assessment of helical tomotherapy plans complexity

**DOI:** 10.1002/acm2.13781

**Published:** 2022-12-15

**Authors:** Samuele Cavinato, Marco Fusella, Marta Paiusco, Alessandro Scaggion

**Affiliations:** ^1^ Medical Physics Department Veneto Institute of Oncology IOV‐IRCCS Padova Italy; ^2^ Dipartimento di Fisica e Astronomia “G. Galilei” Università degli Studi di Padova Padova Italy

**Keywords:** complexity metrics, helical tomotherapy, patient‐specific quality assurance

## Abstract

**Purpose:**

An unnecessary amount of complexity in radiotherapy plans affects the efficiency of the treatments, increasing the uncertainty of dose deposition and its susceptibility to anatomical changes or setup errors. To date, tools for quantitatively assessing the complexity of tomotherapy plans are still limited. In this study, new metrics were developed to characterize different aspects of helical tomotherapy (HT) plans, and their actual effectiveness was investigated.

**Methods:**

The complexity of 464 HT plans delivered on a Radixact platform was evaluated. A new set of metrics was devised to assess beam geometry, leaf opening time (LOT) variability, and modulation over space and time. Sixty‐five complexity metrics were extracted from the dataset using the newly in‐house developed software library TCoMX: 29 metrics already proposed in the literature and 36 newly developed metrics. Their reciprocal relation is discussed. Their effectiveness was evaluated through correlation analyses with patient‐specific quality assurance (PSQA) results.

**Results:**

An inverse linear relation was found between the average number of closed leaves and the average number of MLC openings and closures as well as between the choice of the modulation factor and the discontinuity of the field, suggesting some intrinsic link between the LOT distribution and the geometrical complexity of the MLC openings. The newly proposed metrics were at least as correlated as the existing ones to the PSQA results. Metrics describing the geometrical complexity of the MLC openings showed the strongest connection to the PSQA results. Significant correlations were found between at least one of the new metrics and the *γ* index passing rate $PR_{\gamma }\%\left(3\%G,2\mathrm{mm}\right)$ for six out of seven groups of plans considered.

**Conclusion:**

The new metrics proposed were shown to be effective to characterize more comprehensively the complexity of HT plans. A software library for their automatic extraction is described and made available.

## 1 INTRODUCTION

Helical tomotherapy (HT) is among the most advanced technologies nowadays available for external beam radiotherapy. During an HT treatment, beam fluence modulation is achieved through a binary MLC that acts on a fan‐beam that rotates around the patient, whereas the couch is translated through the gantry at a constant speed. Thousands of beamlets are typically involved in this process, a fact that makes this technique very suitable to deliver a highly conformal dose to the PTV and optimal OARs sparing at the same time.

This high degree of dynamic modulation of machine parameters, commonly referred to as plan complexity, places high demands on treatment machines and TPSs. For intensity‐modulated radiation therapy (IMRT) and for volumetric modulated arc therapy (VMAT) delivered on C‐arm linacs,^1^ high degree of complexity augments the uncertainty related to treatment delivery and dose calculation, and also increases the sensitivity of the delivered dose to small deviation in machine parameters and in the patient's geometry.^2^, ^3^, ^4^, ^5^, ^6^ Such relation led to the introduction of several indexes, the so‐called complexity metrics, to quantify factors that may impact the quality and deliverability of IMRT/VMAT treatments. Such metrics should allow one to precisely quantify and control the degree of modulation characterizing each treatment plan, which can be helpful to evaluate trade‐offs between dosimetric performance and plan complexity and can also serve as a plan verification tool aimed at reducing the quality assurance (QA) workload if used to build a predictive model of the patient‐specific QA (PSQA).^7^, ^8^, ^9^

The extension of the complexity metrics designed for VMAT to HT is not straightforward due to the intrinsic differences in the delivery modality. Several studies in the literature investigated the role of typical tomotherapy delivery parameters such as the modulation factor (MF), the maximum and mean leaf opening time (LOT) as well as individual leaf latencies^10^, ^11^, ^12^ as possible indicators of complexity. More precisely, they tried to propose action ranges on these parameters as well as operative rules to support the planner in the realization of clinically deliverable plans, namely, plans with $PR_{\gamma }\%\left(3\%G,2\mathrm{mm}\right)$ > 95%.^13^ Despite the usefulness of having practical indications during the planning of the treatment, the proposed strategies do not allow a detailed measure of an HT plan complexity. Having a set of robust complexity indicators can facilitate the plan comparison and has the potential to drive a standardization of the optimization approach so as to reduce the inter‐ and intra‐planner variability and share effective planning strategies across the community.

Recently, Santos et al.^14^ proposed a set of complexity metrics specifically designed for HT. The authors proved that this set of metrics provide a quantitative description of the differences among three categories of treatment that were known in advance to exhibit qualitative differences in the plans’ sinograms (i.e., stereotactic treatments of the brain, prostate plans, and head and neck plans). Some of the indexes showed mild correlation with PSQA results of stereotactic brain plans only. Santos et al.^14^ provided a first indication of how the HT plan complexity can be quantified and paved the way for a comprehensive analysis of the relationship between HT plan complexity and HT plan deliverability.

The main goal of the present work is to reduce the gap toward the definition of a robust group of indicators capable of predicting possible PSQA failures by systematically extending the current set of available metrics for the characterization of HT plans complexity. To this purpose, a new set of complexity metrics was devised to provide quantitative information on four fundamental aspects of HT treatment plans: statistics of the LOT distribution, geometrical properties of the binary MLC openings related, for example, to the area and the discontinuity of the field, inter‐projection and inter‐leaf LOT variability, and modulation of the leaf openings over space and time. In addition to that, some of the existing indexes were redesigned to provide them with more flexibility and increase their descriptive power. Metrics were extracted using the newly in‐house developed software library *TCoMX* that is made available. We investigated if the newly proposed complexity metrics allow us to deepen the understanding of HT plans properties and if they grant an increased predictive capability of PSQA result when compared to the metrics already existing in the literature.

## 2 MATERIALS AND METHODS

### 2.1 Dataset

Between June 2018 and April 2021, a total of 464 treatments have been planned with the Precision TPS v1.0.02 (Accuray, Inc., Madison, Wisconsin, USA) using the GPU‐based optimizer VOLO, which incorporates the Collapsed Cone Convolution Superposition dose calculation algorithm.^15^, ^16^ The dataset used in this work is an extension of the one used in Ref. [17]. The treatments were related to different sites and diseases. They have been grouped in the following categories: brain, head and neck (HN), thorax, abdomen, pelvis, prostate, and others. The dose per fraction (*D*/*fr*) ranged from 1.5 to 5 Gy. For all the plans, dedicated PSQA sessions were performed, and two different *γ* index passing rates ($PR_{\gamma }\%$) were collected: 3% global DD, 2 mm DTA ‐ $PR_{\gamma }\%\left(3\%G,2\mathrm{mm}\right)$ ‐ used in the clinical routine^13^ and, as reported in previous works,^18^, ^19^ the more sensitive 2% local DD, 2 mm DTA ‐ $PR_{\gamma }\%\left(2\%L,2\mathrm{mm}\right)$ ‐ to highlight delivery errors. Both were computed with a 10% dose threshold.^20^ The PSQA measurements were performed with the ArcCHECK detector array (Sun Nuclear Corporation, Melbourne, FL, USA) without making use of the PMMA CavityPlug. The plans were recomputed on the homogeneous synthetic ArcCHECK CT (density of 1.1836 g/cm^3^) in high‐resolution mode (grid size 1.87 mm). Acquisition, analysis, and calculation of $PR_{\gamma }\%$ were performed with Sun Nuclear SNC Patient version 6.7.

### 2.2 Complexity metrics

In this section, all the metrics considered in this work are reported. Furthermore, a detailed description of the newly developed ones is presented. A summary of all the new and old metrics considered is provided in Table 1. A list with all the acronyms and abbreviations used in the work can be found in Table S1.

**TABLE 1:** List of the metrics extracted in the work

Category	Subcategory ↓/Group→	Old (29)	New (36)
TPS	Delivery	Pitch, FW, PT, GP, TT, TL, CS, CT, *N_proj_*, *N_rot_*, MF, TTDF	
LOT statistics	Absolute LOT	mLOT, sdLOT, mdLOT, moLOT, minLOT, maxLOT, CLNS_100_, CLNS_50_, CLNS_30_, CLNS_pt,20_,	kLOT, sLOT, CLNS_20_,
	Relative LOT		mFLOT, sdFLOT, moFLOT, mdFLOT, minFLOT, maxFLOT, CFNS_5_, CFNS_10_, CFNS_50_, CFNS_75_, CFNS_90_
Sinogram	Geometry	L0NS, L1NS, CLS, *CLS_in_*	L2NS, *nCC*, lengthCC, TA, fDISC, *CLS_in,area_*, *CLS_in,disc_*, *CLS_in,area,disc_*, centroid
	Modulation	PSTV, LOTV, MI	nOC, EPSTV_1,1_, EPSTV_0,1_, EPSTV_1,0_, ELOTV_1_, ELOTV_2_, ELOTV_3_, ELOTV_4_, ELOTV_5_, mSI, mdSI, sdSI, MSA

#### 2.2.1 TPS parameters

This category contains the typical parameters considered during the planning process and that can be tuned directly (i.e., by the planner) or indirectly (i.e., by the optimizer) in the TPS through the optimization process. These are all grouped in a single subcategory called *Delivery* that gathers the following: modulation factor (MF), projection time (PT), gantry period (GP), treatment time (TT), target length (TL), couch translation (CT), couch speed (CS), number of projections (*N_proj_*), number of rotations (*N_rot_*), pitch, field width (FW), and the ratio between the TT and the fraction dose. We refer to the existing literature for the mathematical definitions and to the *TCoMX User Manual* ^21^ for the details concerning their computation.

#### 2.2.2 LOT statistics

This category contains a selection of statistical metrics that can be used to quantitatively describe the LOT distribution. Two subcategories were identified.

*Absolute LOT statistics*. The statistical metrics describing the absolute LOT distribution are: mean (mLOT), median (mdLOT), mode (moLOT), standard deviation (sdLOT), kurtosis (kLOT), skewness (sLOT), maximum (maxLOT), minimum (minLOT), fraction of LOTs smaller than *n* ms (here called cumulative LOT number score, CLNS*_n_*, also called LOT*_n_* in previous works), and percentage of LOTs within *n* ms from the PT (here called cumulative LOT number score at PT, CLNS_pt,_ *_n_*, also called LOTpt*N* in previous works).

*Relative LOT statistics*. We introduced de novo a group of statistical quantities describing the fractional LOT (FLOT) distribution, namely, the absolute LOT distribution normalized by the PT. They are: mean (mFLOT), median (mdFLOT), mode (moFLOT), standard deviation (sdFLOT), maximum (maxFLOT), minimum (minFLOT), percentage of FLOTs smaller than *n*, with n∈[0,1] (named here cumulative FLOT number score, CFNS_nx100_). Although most of them are related to the corresponding absolute LOT statistics through the PT, FLOT statistics have been introduced in this work to allow an easier and more meaningful comparison among plans with large differences in PT.

#### 2.2.3 Sinogram

Complexity metrics can be extracted directly from the sinogram to describe different features concerning the geometry of the field and the modulation and variability of the LOTs. Thereby, two main subcategories were identified.

##### Geometry

They describe the geometry of the field at each projection. They aim at describing the discontinuity of the field, its area, and amount of edges with the aim of quantifying aspects related to the penumbra effects of the MLC as well as to the geometrical complexity and size of the field at the different projections. The metrics already found in literature^14^ and belonging to this subcategory are the open leaves with *n* open neighbors score (LnNS) and the closed leaf scores, namely, the CLS and *CLS_in_*. In addition to them, new metrics have been developed in this work, and they are described in the following.

*Treatment area (TA)*. It is defined as follows:

(1) $$TA=\frac{1}{N}\sum_{i\;=\;1}^{N}\left|R_{i}-L_{i}\right|+1$$

where *N* is the total number of projections, while $R_{i}$ and $L_{i}$ are the indexes of the rightmost and leftmost open leaves at projection *i*, respectively. The plan value is obtained by averaging over *N*. As the LOT values are not considered in this definition, TA represents the average open area. The treatment area was already defined by Santos et al.^14^ for the computation of the *CLS_in_*, but it was not considered as an independent metric itself.

*Centroid (C)*. It is defined as follows:

(2) $$C=\frac{1}{N}\sum_{i\;=\;1}^{N}\frac{1}{\sum_{j\;=\;1}^{L}O_{ij}}\sum_{j\;=\;1}^{L}O_{ij}P_{j}$$

where $O_{ij}$ is the so‐called mask‐sinogram, namely, a matrix with the same size of the sinogram whose entries are set to 0 for all (F)LOTs = 0, and 1 in all other cases. $P_{j}$ is the so‐called Leaf Position Array, namely, an (1×L)‐dimensional array that contains the indexes representing the mid position of each leaf in dimensionless units and taken from the geometrical center of the MLC (e.g., ±1 and ±2). The centroid is measured in number‐of‐leaves. The plan value is obtained by averaging over *N*. The centroid of a plan represents the mean of the average positions of the open leaves at each projection. This metric should highlight geometrical asymmetries of the sinogram arising when noncentral targets are irradiated and which might increase the overall geometrical complexity of the treatment.

*Number of connected components (nCC)*. It is a dimensionless metric that counts the number of independent groups of connected open leaves inside the treatment area. It is computed at each projection and then averaged over all the projections to get the plan value. In Figure S1, an example of two projections with different numbers of connected components is shown.

*Length of the connected components (lengthCC)*. It is defined as follows:

(3) $$lengthCC=\frac{1}{\sum_{i\;=\;1}^{N}nCC_{i}}\sum_{i\;=\;1}^{N}\sum_{k\;=\;1}^{nCC_{i}}\left|L_{k}-R_{k}\right|+1$$

where $L_{k}$ and $R_{k}$ are indexes representing the positions of the leftmost and rightmost open leaves of the *k*th connected component at the *i*th projection, respectively. It is measured in number‐of‐leaves. The plan value measures the average length of the connected components. We refer the interested reader to Figure S1 for graphical representation.

*Fraction of discontinuous projections (fDISC)*. It is a dimensionless metric defined as follows:

(4) $$fDISC=\frac{1}{N}\sum_{i\;=\;1}^{N}\left[nCC_{i}>1\right]$$

where the term $\left[nCC_{i}>1\right]$ is 0 if $nCC_{i}=0\;\mathrm{or}\;1,$ 1 otherwise. It counts the fraction of projections with two or more connected components that are all the projections having at least one closed leaf within the treatment area.

*Closed leaf scores within the treatment area (CLS_in_)*. The *CLS_in_* was introduced in Ref. [14] with this definition:

(5) $$CLS_{in}=\frac{1}{N}\sum_{i\;=\;1}^{N}\frac{TA_{i}-\sum_{j\;=\;1}^{L}O_{ij}}{L}$$

A more general form has been devised, and three variants have been introduced:

- $CLS_{in,area}$: The number of closed leaves within the treatment area at projection *i* is normalized by $TA_{i}$ instead of *L*.
- $CLS_{in,disc}$: It is computed by considering the discontinuous projections ($nCC_{i}>1)$ only, namely, the first summation in Equation (5) runs from 1 to the total number of discontinuous projections and is then divided by the number of discontinuous projections instead of by *N*.
- $CLS_{in,area,disc}$: It corresponds to the combination of the $CLS_{in,area}$ with the $CLS_{in,disc}$. The three quantities are in general correlated with each other. However, the different definitions should help to characterize the geometry of the leaf openings in a more intuitive way.

##### Modulation

These metrics describe the modulation and variability of the HT fan‐beam over time and across leaves. They are computed by combining the (F)LOTs at different projections and/or leaves. All the metrics belonging to this subcategory aim at describing the variability of the LOTs across the different projections.

*Extended leaf open time variability (ELOTV_Δp_)*. It is a dimensionless metric defined as follows:

(6) $$ELOTV_{\Delta p}=\frac{1}{L}\sum_{j\;=\;1}^{L}\frac{\sum_{i\;=\;1}^{N-\Delta p}\left|S_{ij}-S_{i+\Delta p,\;j}\right|}{\left(N-\Delta p\right)\times \left(S_{j}\right)}$$

where Δp is the projection step, namely, the distance between the projections considered, $S_{ij}$ the element (*ij*) of the sinogram, and $S_{j}$ the *j*th column (leaf) of the sinogram. It takes values in [0;1], being 0 when all the leaves have the same opening time at each projection. The $ELOTV_{\Delta p}$ is first evaluated for each leaf and then averaged over all the leaves to obtain the plan value. For the leaves that do not open during the treatment, it is set to 0 by definition. It is worth noticing that the $ELOTV_{\Delta p}$ includes the LOTV introduced in Ref. [14] as a special case. In particular,

(7) $$LOTV=1-ELOTV_{1}$$

Compared to the LOTV, the $ELOTV_{\Delta p}$ shows two main differences: It is positively correlated with the inter‐projection (F)LOT variability and allows the comparison of projections that lie at arbitrary distances. Despite one might expect $ELOTV_{\Delta p}$ to be highly correlated with the LOTV, the freedom in the choice of the projection step Δp might allow to highlight further properties of the sinogram.

*Extended plan sinogram time variation (EPSTV_Δp,Δl_)*. It is a dimensionless metrics defined as follows:

(8) $$EPST\;V_{\Delta p,\;\Delta l}=\frac{1}{N-\Delta p}\sum_{i\;=\;1}^{N-\Delta p}\sum_{j\;=\;1}^{L-\Delta l}\left|S_{i+\Delta p,j}-S_{i,j}\left|+\right|S_{i,j+\Delta l}-S_{i,j}\right|$$

where Δl is the leaf step, namely, the distance between the two considered leaves. The $EPSTV_{\Delta p,\;\Delta l}$ contains the PSTV defined in Ref. [14] as a special case, namely,

(9) $$PSTV=EPSTV_{1,1}$$

The formulation in Equation (8) allows the comparison of projections/leaves that lie at arbitrary distances. Furthermore, there are two special cases included in Equation (8), which are worth mentioning:

1. $EPSTV_{0,\;\Delta l}$: time variation along the leaves direction only;
2. $EPSTV_{\Delta p,\;0}$: time variation along the projection direction only.

In general, a higher $EPSTV_{\Delta p,\;\Delta l}$ corresponds to higher inter‐leaf and/or inter‐projection (F)LOT variabilities. Similarly to what was stated for the $ELOTV_{\Delta p}$, the freedom in the choice of both the projection and leaf steps allows a broad spectrum of investigation into the properties of the sinogram.

*Number of openings and closures (nOC)*. It is a dimensionless metric, and it is computed by counting the number of times each leaf opens and closes during the treatment. The plan value is obtained by averaging over the leaves and normalizing by the total number of projections.

The number of openings and closures is computed considering that each (F)LOT is centered with respect to the PT.^11^ In Figure S2, a schematic of the different conditions that might be encountered during the treatment is shown.

This metric is associated with the mechanical stress of the MLC during the treatment and might also be connected to the latencies of the leaves. Moreover, this metric is connected to the CLS (average fraction of closed leaves per projection) by the following approximate linear relationship:

(10) CLS≈1−0.5×nOC

This is due to the fact that fully closed leaves ($S_{ij}=0)$ have $nOC_{ij}=0$ and fully open leaves ($S_{ij}=1)$ appear only in a negligible amount. Therefore, each $0<S_{ij}<1$ correspond to $nOC_{ij}=2$.

*Mean sinogram asymmetry (MSA)*. It is defined as follows:

(11) $$MSA=\frac{\sum_{j=1}^{L}P_{j}\times LPS_{j}}{\sum_{j=1}^{L}LPS_{j}}$$

where $LPS_{j}$ is called *Leaf Projected Sinogram*, and it is defined as

(12) $$LPS_{j}=\sum_{i=1}^{N}\frac{S_{ij}}{N}$$

An example of LPS is shown in Figure S3. The MSA represents the weighted average displacement of the (F)LOTs from the vertical axis passing through the center of the sinogram, and it is measured in number‐of‐leaves. It allows us to highlight asymmetries of the sinogram that might be related to an increasing complexity in the presence of noncentral targets.

*Sinogram intensity*. Starting from the LPS defined in Equation (12), it is possible to compute another set of metrics. In particular, we considered the mean sinogram intensity (mSI), computed as the average of the LPS values over the MLC leaves. In addition, also the standard deviation (sdSI) and the median (mdSI) were computed.

### 2.3 Metrics extraction

A set of 65 metrics was extracted from the dataset. Within the scope of the analysis performed in this study, they were subdivided into two groups: The existing metrics reported in the literature (*Old*), and a set of metrics chosen among the new ones proposed in this study by setting the different parameters described before (*New*). The two groups are composed of 29 and 36 metrics, respectively. A comprehensive list is reported in Table 1. In the case of the $CLNS_{n}$, n={20,30,50,100} were chosen, whereas for the CFNS_nx100_, n={0.05,0.10,0.5,0.75,0.9} were set.

All the possible variants of the LnNS and CLS were computed, whereas a choice was made for the $ELOTV_{\Delta p}$ and $EPSTV_{\Delta p,\Delta l}$. The former was computed for Δp∈[1,5] to show the behavior one should expect when the projection step is increased. The case Δp=1 was chosen to show the relation in Equation (7) explicitly. The $EPSTV_{\Delta p,\Delta l}$ was computed for three different combinations of (Δp,Δl), namely, (1,1),(1,0),(0,1). The first pair of parameters was chosen to show the relation in Equation (9) explicitly, whereas the other two in order to consider only the projection and leaf directions, respectively.

### 2.4 The TCoMX library

The TCoMX (Tomotherapy Complexity Metrics EXtractor) library^22^ (github.com/SamueleCavinato/TCoMX) is an in‐house developed Matlab (The MathWorks Inc, Natick, MA, USA) library for the automatic extraction of a wide set of complexity metrics from the DICOM RT‐plan files of HT. These metrics are not explicitly stored in this file, and all the computations are performed starting from the plan's sinogram, which is instead stored in it. It was proposed and used for the first time in this work. The current version of TCoMX (v1.0) allows the extraction of all the different complexity metrics reported in Section 2.2, some of them with customizable parameters.

TCoMX is compatible with DICOM RT‐plans files generated using both RayStation (RaySearch Laboratories, Stockholm, Sweden) and Precision (Accuray, Sunnyvale, CA) TPSs. A reference dataset composed by 18 anonymized RT plans (9 Precision, 9 RayStation) is also provided in the repository. For all the technical details concerning TCoMX, we refer to the *TCoMX User Manual*.^21^

The use of this library may help to standardize the metrics extraction process and make the comparison of the results obtained across different centers more robust.

### 2.5 Statistical analysis

#### 2.5.1 Correlations among metrics

To determine, the reciprocal relation between the different aspects described by the whole bunch of available metrics, a correlation analysis was performed. This allowed us to highlight some possible intrinsic dependences among properties of tomotherapy plans and investigate the mutual relation between the whole set of extracted metrics. A correlation analysis was performed on the whole dataset without any distinction among groups of plans, and both the inter‐ and intra‐subcategory correlations were analyzed. Spearman's correlation coefficient was chosen as the indicator of correlation, and the significance level was set to 0.05. As generally accepted, the correlation values were classified into five classes: very weak (0≤|r|<0.2), weak (0.2≤|r|<0.4), moderate (0.4≤|r|<0.6), strong (0.6≤|r|<0.8), and very strong (|r|≥0.8).

Following the analysis by Boyd et al.,^23^ we tried to understand if plans with different degrees of delivery efficiency showed different patterns of complexity and modulation. To this extent, two independent subsamples were extracted from the whole dataset on the basis of CFNS_75_ value. Plans with CFNS_75_ < 25th percentile represent the most efficient plans, whereas plans with CFNS_75_ > 75th percentile represent the least efficient ones. The two groups are compared through a Student's *t*‐test.

#### 2.5.2 Relation between the metrics and the PSQA results

The relation between the metrics and the results of PSQA analysis was investigated through a correlation analysis. This analysis was performed for each of the seven categories of plans independently. Additionally, the whole dataset was considered too. The metric‐$PR_{\gamma }\%$ Spearman's correlation matrix, $\rho_{metric,\;PR_{\gamma \;}\%}$, was computed for each of the two considered PRγ%. Only the metrics that turned out to be significantly correlated with the $PR_{\gamma }\%$ $\left(p_{value}\;\left(\rho_{metric,\;PR_{\gamma \;}\%}\right)<0.05\right)$ were kept. Furthermore, if two metrics were (very) strongly correlated to each other, the one with the smallest $|\rho_{metric,\;PR_{\gamma \;}\%}|$ was removed. The number of metrics selected from each of the two groups after this procedure was collected and compared.

## 3 RESULTS

### 3.1 Correlation among metrics

**FIGURE 1:**
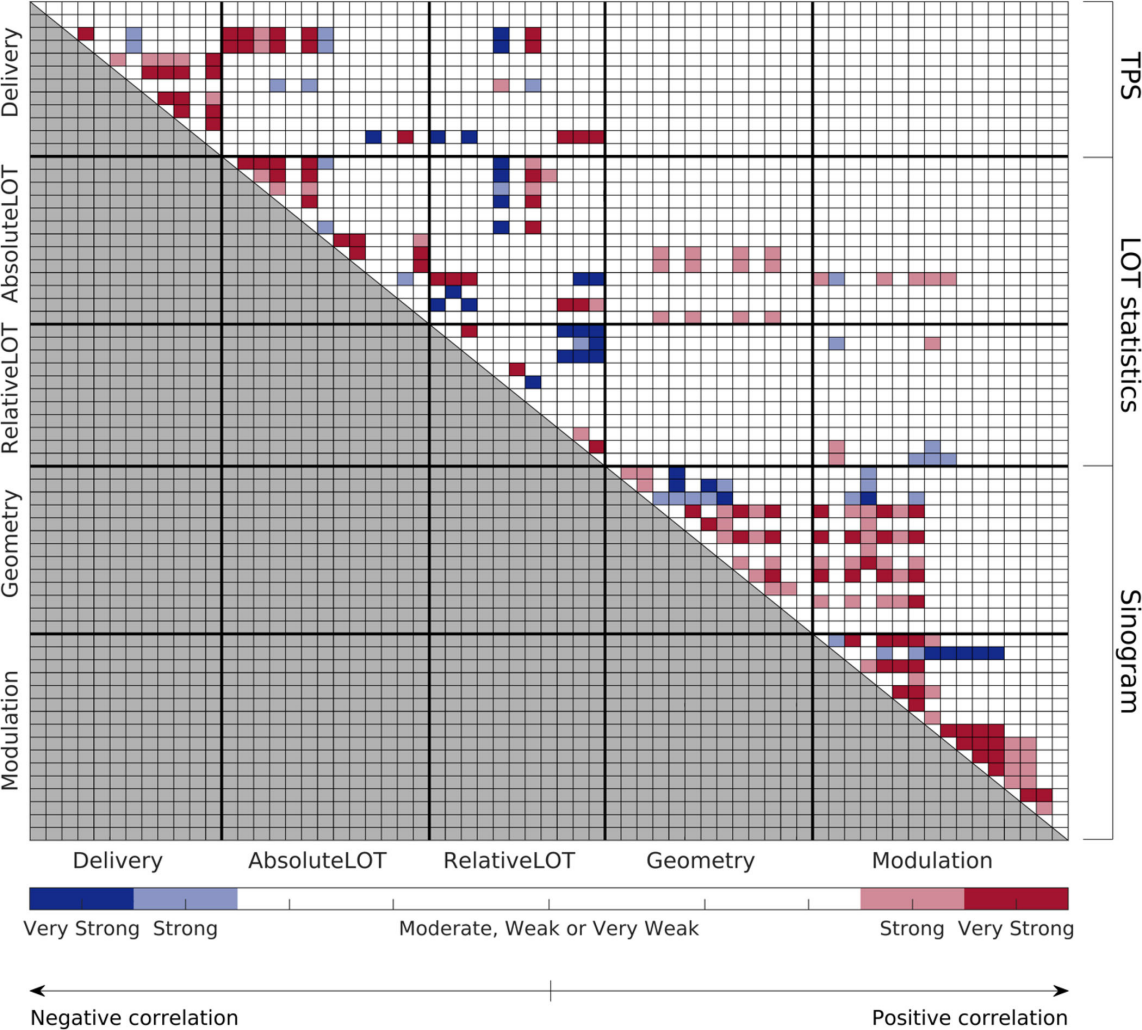
Full Spearman's inter‐subcategory correlation map. Only strong and very strong correlations are reported. The black thicker solid lines mark the separation between the subcategories.

In Figure 1, the full metric–metric correlation map is shown. All the five subcategories introduced before are considered, and the metrics in each subcategory are sorted as in Table 1. Only strong or very strong correlations are purposely shown. The two clusters of metrics composed of *Delivery, Absolute LOT*, and *Relative LOT* on one side and *Geometry* and *Modulation* on the other have shown to be not (very) strongly correlated among themselves.

As expected from Equation (10), a very strong negative correlation between nOC and CLS (r=1) was found, which confirms the linear relation between the quantities. Strong correlations were found between MF and CLNS_pt,20_ (r=−0.81), between (MI, EPSTVs) and the *CLS_in_* (r>0.74), and between the L1NS and CLS (r=0.76).

**FIGURE 2:**
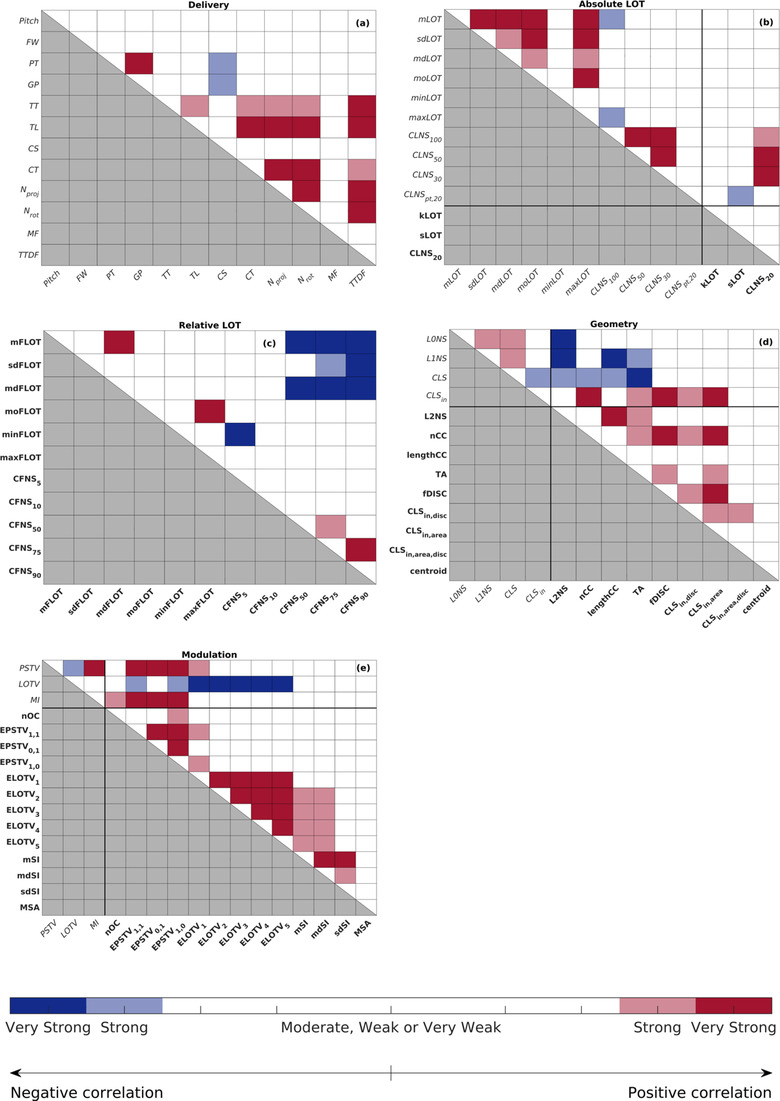
Significant Spearman's correlations among the metrics in each of the five different subcategories considered. Metrics belonging to the New and Old group are marked using the bold and italic font, respectively. The black thicker solid lines mark the separation between the two groups.

In Figure 2, the intra‐subcategory correlations are shown. Among all, it is interesting to observe that the analysis of the geometrical properties of the sinogram shows that TA is at least strongly correlated with most of the metrics describing the discontinuity of the projections: the different variants of the CLS (r=−0.98), *nCC* (r=0.68), fDISC (r=0.65), and L1NS (r=0.66). No (very) strong correlations were found instead between the centroid and the other geometrical metrics.

Three groups of strongly correlated metrics arose in the subcategory *Modulation*: the EPSTVs, the LOTVs, and the SIs. A strong positive correlation was found between MI and nOC (r=0.63). No strong correlations were found between the MSA and any other metric.

**FIGURE 3:**
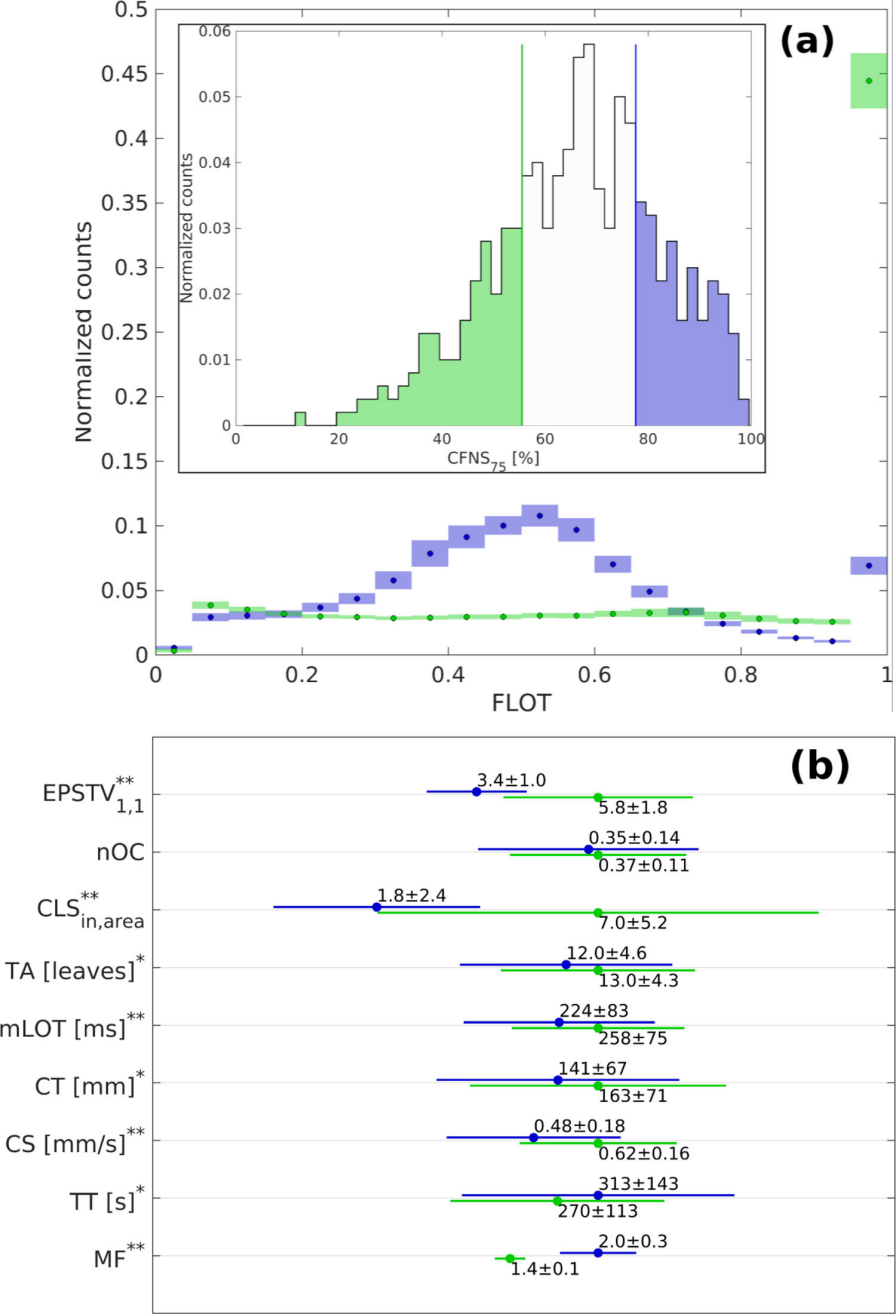
(a) [Inner panel] Distribution of the CFNS_75_ over the whole dataset of 464 plans. Data corresponding to most (least) efficient plans are highlighted in green (blue). Vertical lines represent the 25th and 75th percentile. [Outer panel] Average FLOT histogram for the two subsamples. Points mark the mean number of (normalized) counts for each bin and the colored boxes represent the 95% Gaussian CI around it. (b). Mean values and standard deviations of the nine metrics selected. Green (blue) lines and markers refer to the most (least) efficient plans. The symbol * (**) near the metrics’ names indicates statistically significant comparisons according to a Student's *t*‐test at 0.05 (0.01) significance level.

In Figure 3a, the average FLOT distributions obtained for the two subsamples corresponding to the least and most efficient plans, respectively, are shown. Figure 3b shows the mean values of nine representative metrics selected from the different subcategories. The values for all the other metrics as well as the *p*‐values of Student's *t*‐test are reported in Tables S2–S6. The least efficient plans showed significantly higher values of MF and TT and this resulted in significantly smaller values of EPSTV_1,1_, *CLS_in,area_*, TA, mLOT, TL, and CS. No statistical differences arose for the number of openings and closures nOC.

### 3.2 Relation between the metrics and the PSQA results

**FIGURE 4:**
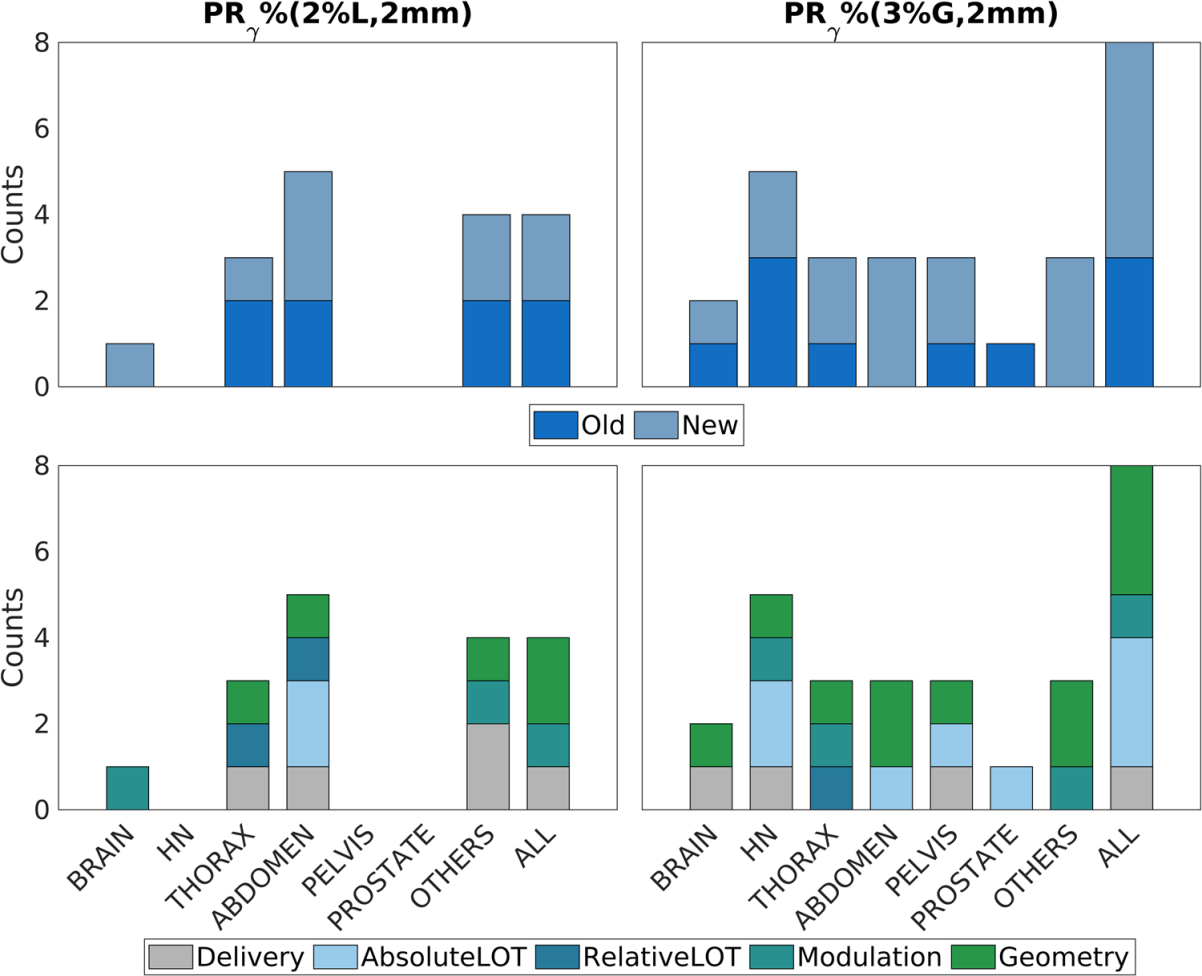
Barplots showing the final number of metrics (*y* axis) correlated to the patient‐specific quality assurance (PSQA) results after the selection procedure for each of the groups of plans considered (*x* axis). (a and b) Separation between Old and New metrics is highlighted. (c and d) Separation between the subcategories is highlighted.

In Figure 4a,b, the numbers of metrics selected from the two groups of metrics (*Old* and *New*) for each of the seven groups of plans and for the whole dataset are shown. At least one significant correlation was found with $PR_{\gamma }\%\left(3\%G,2\mathrm{mm}\right)$ for each group of plans. No significant correlations were found between the metrics and the $PR_{\gamma }\%\left(2\%L,2\mathrm{mm}\right)$ for HN, pelvis, and prostate.

Figure 4c,d shows the same results of Figure 4a,b, but the different metrics subcategories are now highlighted. Metrics belonging to *Geometry* appear in 11 out of the 13 cases where significant correlations were found, 8 for the $PR_{\gamma }\%\left(3\%G,2\mathrm{mm}\right)$ and the remaining 5 for the $PR_{\gamma }\%\left(2\%L,2\mathrm{mm}\right)$. *Geometry* is followed by *Delivery*, *Modulation*, *Absolute LOT*, and *Relative LOT* in descending order.

In Table 2, the metrics with the highest correlation with each of the two $PR_{\gamma }\%$ for each group of plans and for all the plans are reported. Seven out of thirteen metrics belong to the group *New*. In general, the correlation values span between weak and moderate, with one case of strong correlation between the MSA and the $PR_{\gamma }\%\left(2\%L,2\mathrm{mm}\right)$ for the brain.

**TABLE 2:** Highest Spearman's correlation coefficients between the metrics and the patient‐specific quality assurance (PSQA) results

	$PR_{\gamma }\%\left(2\%L,2\mathrm{mm}\right)$		$PR_{\gamma }\%\left(3\%G,2\mathrm{mm}\right)$	
Group	Metric	ρ	Metric	ρ
Brain	**MSA**	0.63	**CLS_in,disc_**	0.46
HN			**fDISC**	0.34
Thorax	L0NS	−0.36	L0NS	−0.25
Abdomen	mLOT	−0.49	**nCC**	0.47
Pelvis			TTDF	0.25
Prostate			minLOT	0.33
Others	FW	0.55	**CLS_in,area,disc_**	0.48
All	**sdSI**	0.21	**TA**	0.33

*Note*: Metrics belonging to the New and Old group are marked using the bold and italic font, respectively.

## 4 DISCUSSION

In this study, a new set of complexity metrics designed specifically for HT was proposed. They are organized into categories and subcategories according to the feature of the plan they describe. They are computed with the in‐house developed Matlab library TCoMX freely available online. Sixty‐five of these metrics were extracted from the dataset here considered.

The analysis of the Spearman's correlation among the metrics confirmed some of the results already reported in the literature, in particular the very strong correlations among the CLNS*_n_* s, the strong negative one between MF and the CLNS_pt,20_, the one between MI and PSTV and the *CLS_in_*, and the one between the L1NS and CLS.^10^, ^12^, ^14^

However, the introduction of a set of newly developed complexity metrics allowed us to focus on further aspects of the HT treatment plans that have never been, or only partially, considered before. In particular, the introduction of the *Relative LOT statistics*, and more precisely the CFNS_75_, allowed us to identify two subsamples of plans characterized by different characteristics LOT distribution's shape and treatment's efficiency. These results closely match the idea proposed by Boyd et al.^23^: The least efficient plans (those with CFNS_75_ > 75th percentile) exhibit an average bimodal LOT distribution with a first peak around FLOT = 0.5 and a second one around FLOT = maxFLOT. On the other hand, the most efficient plans (those with CFNS < 25th percentile) show an overall flat trend characterized by a marked peak in the last bin.

The modulation of delivery of the most efficient plans, namely those with small values of CFNS_75_, is achieved mainly by acting on the geometry of the beam by splitting the field at each projection into two or more disjoint subfields. This results in a higher number of in‐field closed leaves (higher *CLS_in,area_*), as well as a generally higher number‐of‐leaves involved (higher TA). Such plans showed shorter and faster treatments (smaller TT and higher CS), despite the significantly longer translation of the couch (CT). On the other hand, the field at each projection was found to be less disjoint for the least efficient plans, namely those with high values of CFNS_75_, so that the modulation seems to be mainly achieved by acting on the LOTs values (i.e., higher MF). The two groups of plans do not exhibit significant differences in the FW, making all the previous conclusions independent from it. Furthermore, no significant differences were observed in the PSQA results, suggesting that a future implementation of the indicators proposed in this work in the optimization process might help to get efficient plans without affecting the deliverability.

In general, the newly proposed metrics (belonging to the *New* group) have been shown to be at least as correlated to the PSQA results ($PR_{\gamma }\%$) results as the existing ones. At least one of them appears for each of the considered groups of plans for whom significant correlations arose, apart in one case ($PR_{\gamma }\%\left(3\%G,2\mathrm{mm}\right)$ for prostate). More precisely, the newly introduced metrics have shown to be generally more correlated with the ($PR_{\gamma }\%\left(3\%G,2\mathrm{mm}\right)$ where 19 out of the 29 metrics kept after the selection procedure belong to this group. On the other hand, no net difference arose between the *New* and *Old* metrics for the $PR_{\gamma }\%\left(2\%L,2\mathrm{mm}\right)$ where the metrics kept after the selection procedure amount to 8 and 9, respectively.

In three cases ($PR_{\gamma }\%\left(2\%L,2\mathrm{mm}\right)$ for brain, $PR_{\gamma }\%\left(3\%G,2\mathrm{mm}\right)$ for abdomen and others), only metrics pertaining to the *New* group are selected as influential. Although they might be correlated with metrics belonging to the group *Old*, they completely substitute metrics from the group *Old* when considered in the selection process. This suggests a higher predictive power. It is worth observing that all the significant correlations arising for “*others*” need to be taken with care due to the heterogeneity of anatomical districts included in it.

No significant correlations were found between the complexity metrics and the $PR_{\gamma }\%\left(2\%L,2\mathrm{mm}\right)$ for prostate and HN cases, similarly to the results obtained in Ref. [14] for the 3D $PR_{\gamma }\%\left(2\%G,2\mathrm{mm}\right)$. On the other hand, the present study provides the first evidence of a set of complexity metrics specifically designed for HT obtained by combining existing metrics and set of new metrics proposed herein, which show significant correlations with pretreatment QA results for all the groups of plans considered when the $PR_{\gamma }\%\left(3\%G,2\mathrm{mm}\right)$ is used.

Among all the considered metrics, geometrical ones are clearly the most related to the PSQA results. The correlation analysis with the $PR_{\gamma }\%\left(3\%G,2\mathrm{mm}\right)$ showed that they play a prominent role for the majority of the groups of plans, with the most correlated metric belonging to this subcategory for five out of the seven groups of plans (brain, HN, thorax, abdomen, others) as well as for the complete set of plans.

All the geometric complexity indexes give different measures of the discontinuity of the binary MLC openings. However, differently from what might have been hypothesized, the correlations are positive in all cases but one (thorax), suggesting that a higher geometrical complexity of the MLC openings might lead to higher plan deliverability. These results go in the same direction as the ones found in Ref. [14], where strong positive correlations of the CLS and the L1NS with the 3D $PR_{\gamma }\%\left(2\%G,2\mathrm{mm}\right)$ were observed for stereotactic brain plans. However, its interpretation can now be completely reversed. As a matter of facts, we proved the inverse linear relation between CLS and nOC, for example, a larger number of closed leaves imply a smaller number of opening/closing leaves motion. Following existing literature,^11^ we can foster the idea that an increased deliverability might be related to a reduced contribution of leaf latency. As proposed in Ref. [11], even if the impact of small individual leaf errors is not significant^24^, ^25^ their composition over the whole treatment might result in significant deviation in the dose deposition.^26^, ^27^ Relying on the collected data, we are not able to disentangle the two competing effects and specifically designed experiments will be undertaken in the future.

The reported correlations between complexity metrics and PSQA results are strongly related to the clinical practice followed to obtain both the plans and the PSQA results (e.g., Tomotherapy unit, TPS, details of optimization approach, PSQA phantom, and its setup). Therefore, the relevance of the proposed metrics should be grounded and validated through large comprehensive comparisons across different centers. Furthermore, although the new metrics proposed and results obtained provide new intuition concerning possible new strategies to orient the planning process, to date these parameters are not directly accessible by the planner on the TPS during the planning process.

To summarize, we provided four major pieces of evidence. We introduced a new set of HT complexity metrics complementary to the existing ones, suitable to quantify different relevant characteristics of HT plans, and apparently correlated to the plan deliverability and delivery efficiency. The geometrical properties, in particular the discontinuity characteristics of the beam projections (as measured by *CLS_in,disc_*, *CLS_in,area,disc_*, fDISC, and *nCC*), appeared to be the most related to PSQA results, at least in the considered database. The intrinsic relation observed between the beam spatial discontinuity and the number of leaf opening/closing, that is, CLS ∼ −nOC, allowed us to reformulate the conclusions reported by Santos^14^ and enforce impacting role of leaf latency on plan deliverability. Finally, we showed that quantitative FLOTs can discriminate among plans having different levels of delivery efficiency as qualitatively proposed by Boyd et al.^23^

## 5 CONCLUSION

The new sets of complexity metrics proposed in this study have been shown to be at least as correlated with the $PR_{\gamma }\%$ as the existing ones. Furthermore, a group of complexity metrics composed of new and existing metrics showed significant correlations with the $PR_{\gamma }\%\left(3\%G,2\mathrm{mm}\right)$ for all the groups of plans considered herein. Among them, a primary role seems to be played by geometrical metrics. In addition to that, the indicators proposed in this study were shown to be related to the shape of the resulting LOT distribution and to the efficiency of the treatment plans. To our knowledge, this is the first study that explicitly reports about a link among the (F)LOT distribution, the geometry, and modulation of HT treatments, providing an overall understanding of the properties of HT plans and the reciprocal relation among them.

This work does not fully solve the problem of what complexity is and what the actual crucial aspects to consider for the realization of a model to predict the PSQA are. However, it helps to reduce the gap toward its formulation by the introduction of new useful tools, including the library TCoMX, which will simplify and standardize the extraction process and make the comparison across centers more robust.

## FUNDING INFORMATION

This research received funds from “Ricerca Corrente 2022” to cover publication costs.

## AUTHOR CONTRIBUTIONS

SC, AS and MF developed the new set of metrics and conceived the study. SC implemented the whole code and performed the analysis. AS and SC wrote the manuscript with input from all authors. MP supervised the project and provided critical feedback.

## CONFLICT OF INTEREST

The authors have declared no conflict of interest.

## Supporting information



Supporting Information
