# Supplementary material for: Quantitative assessment of helical tomotherapy plans complexity
Source: J Appl Clin Med Phys. 2022 Dec 15;24(1):e13781. doi: 10.1002/acm2.13781 (PMC9860001; doi:10.1002/acm2.13781)
Supplement: Supplementary file 1 — Supporting Information [file ACM2-24-e13781-s001.docx]

**Supplementary material**

**S1. List of acronyms and abbreviations**

*Table S1: List of acronyms and abbreviations used in the manuscript.*

| C | Centroid |
| --- | --- |
| $CLS_{in, area}$ | Closed (inside treatment area) leaf score normalized by TA |
| $CLS_{in, disc}$ | Closed (inside treatment area) leaf score over discontinuous projections |
| $CLS_{in, area, disc}$ | Closed (inside treatment area) leaf score over discontinuous projections normalized by TA |
| $CLS_{in}$ | Closed leaf (inside treatment area) score |
| CLS | Closed leaf score |
| CS | Couch speed |
| CT | Couch translation |
| $CFNS_{n}$ | Cumulative FLOT number score |
| $CLNS_{n}$ | Cumulative LOT number score |
| $CLNS_{pt, n}$ | Cumulative LOT number score at projection time |
| $ELOTV_{\Delta p}$ | Extended leaf open time variability |
| $EPSTV_{\Delta p, \Delta l}$ | Extended plan sinogram time variation |
| FW | Field width |
| maxFLOT | FLOT maximum |
| mFLOT | FLOT mean |
| mdFLOT | FLOT median |
| minFLOT | FLOT minimum |
| moFLOT | FLOT mode |
| sdFLOT | FLOT standard deviation |
| fDISC | Fraction of discontinuous projections |
| FLOT | Fractional leaf opening time |
| $PR_{\gamma}\%$ | gamma passing rate |
| GP | Gantry period |
| LOT | Leaf opening time |
| P | Leaf position array |
| LPS | Leaf projected sinogram |
| lengthCC | Length of the connected components |
| kLOT | LOT kurtosis |
| maxLOT | LOT maximum |
| mLOT | LOT mean |
| mdLOT | LOT median |
| minLOT | LOT minimum |
| moLOT | LOT mode |
| sLOT | LOT skewness |
| sdLOT | LOT standard deviation |
| O | Mask-sinogram |
| MSA | Mean sinogram asymmetry |
| mSI | Mean sinogram intensity |
| mdSI | Median sinogram intensity |
| MF | Modulation factor |
| nCC | Number of connected components |
| nOC | Number of openings and closures |
| $N_{proj}$ | Number of projections |
| $N_{rot}$ | Number of rotations |
| LnNS | Open leaves with n open neighbors score |
| PSQA | Patient-specific quality assurance |
| PT | Projection time |
| S | Sinogram |
| sdSI | Sinogram intensity standard deviation |
| TL | Target length |
| TA | Treatment area |
| TT | Treatment time |
| TTDF | Treatment time over dose per fraction |

**S2. Supplementary figures**


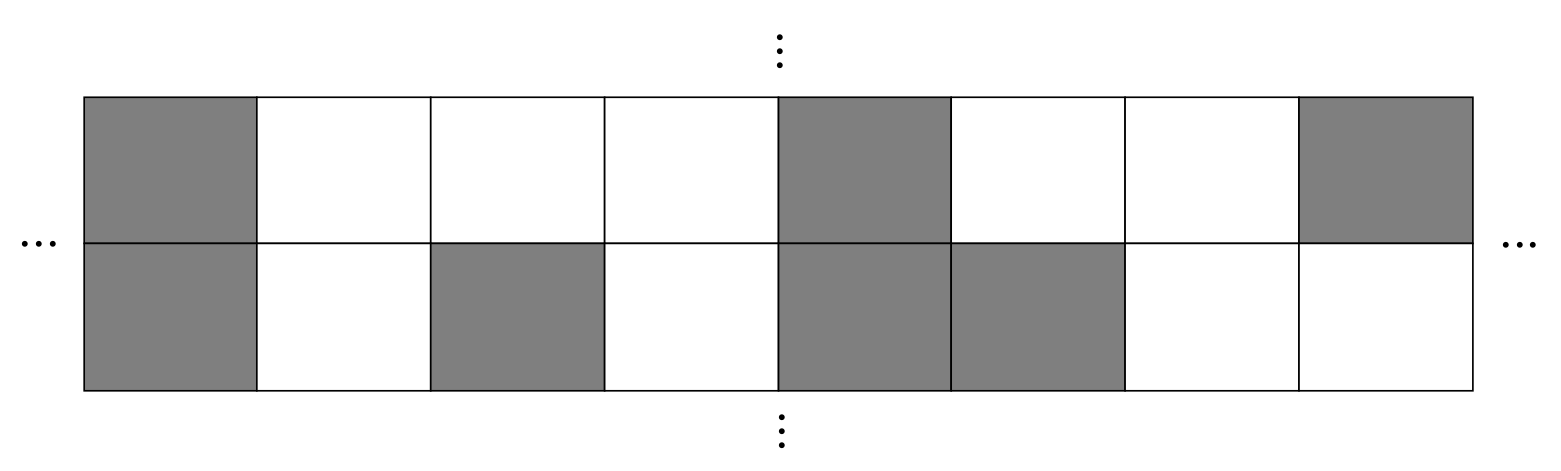


*Figure S1. Schematic representation of two projections with different numbers of connected components. White and grey squares represent open and closed leaves, respectively. The upper projection has 2 connected components, the lower one has 3.*


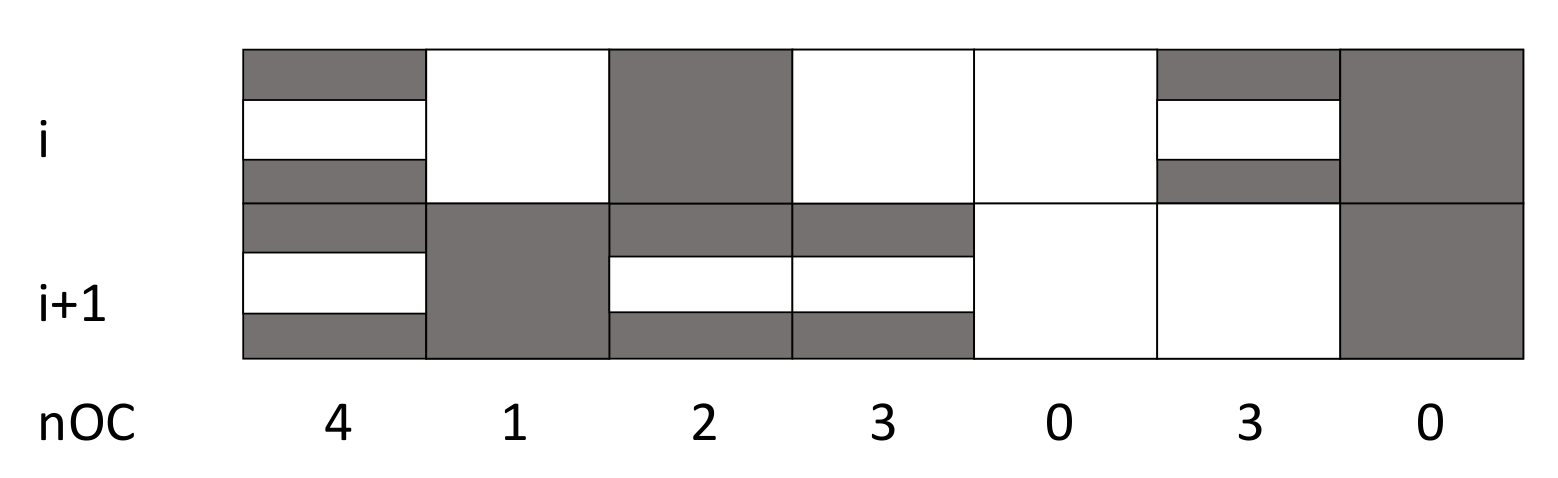


*Figure S2. Schematic representation of possible combinations of the LOTs leading to different numbers of openings and closures. Rows represent two adjacent projections, while columns show seven possible leaf movements. White and gray spaces represent open and closed leaves, respectively. Each LOT is centered about the middle point of the corresponding projection. The number under each leaf represents the cumulative number of openings and closures for each leaf over the two projections.*

*
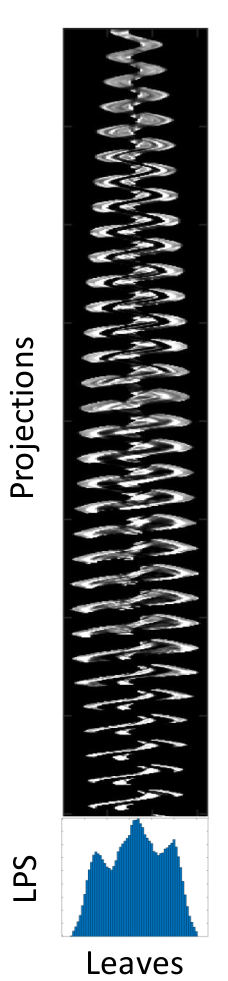
*

*Figure S3. (Upper panel) Example sinogram. Rows correspond to the projections, columns to the leaves. (Lower Panel) Example of LPS. Each bar corresponds to a leaf (column of the sinogram), the height corresponds to the sum over all the projections properly normalized by N, namely the total number of sinogram projections.*

**S3. Values of the metrics for efficient and inefficient plans**

*Table S2: Delivery parameters for the two subsamples at CFNS_75_> 75^th^ percentile and CFNS_75_ < 25^th^ percentile, respectively, and p-value of the t-Student test.*

|  | **CFNS_75_> 75^th^ prctile** | **CFNS_75_ < 25^th^ prctile** | **p-value** |
| --- | --- | --- | --- |
| **Pitch** | 0,38 ± 0,07 | 0,41 ± 0,04 | <0.01 |
| **FW [mm]** | 26,3 ± 5,8 | 26,5 ± 6,2 | 0,79 |
| **PT [s]** | 0,44 ± 0,16 | 0,37 ± 0,11 | <0.01 |
| **GP [s]** | 22,6 ± 8,1 | 18,6 ± 5,8 | <0.01 |
| **TT [s]** | 313 ± 143 | 270 ± 113 | 0,02 |
| **TL [mm]** | 115 ± 64 | 137 ± 70 | 0,02 |
| **CS [mm/s]** | 0,48 ± 0,18 | 0,62 ± 0,16 | <0.01 |
| **CT [mm]** | 141 ± 67 | 164 ± 71 | 0,02 |
| **N_proj_** | 752 ± 385 | 772 ± 327 | 0,67 |
| **N_rotations_** | 15 ± 8 | 15 ± 6 | 0,67 |
| **MF** | 2,0 ± 0,3 | 1,4 ± 0,1 | <0.01 |
| **TTDF [s/cGy]** | 1,4 ± 0,6 | 1,3 ± 0,6 | 0,10 |

*Table S3: Absolute LOT statistics for the two subsamples at CFNS_75_> 75^th^ percentile and CFNS_75_ < 25^th^ percentile, respectively, and p-value of the t-Student test.*

|  | **CFNS_75_> 75^th^ prctile** | **CFNS_75_ < 25^th^ prctile** | **p-value** |
| --- | --- | --- | --- |
| **mLOT [ms]** | 224 ± 82 | 258 ± 75 | <0.01 |
| **sdLOT [ms]** | 96 ± 39 | 115,6 ± 39,9 | <0.01 |
| **mdLOT [ms]** | 219 ± 84 | 302 ± 91 | <0.01 |
| **moLOT [ms]** | 438 ± 160 | 364 ± 113 | <0.01 |
| **kLOT** | 3,4 ± 1,2 | 2,2 ± 0,9 | <0.01 |
| **sLOT** | 0,3 ± 0,5 | -0,7 ± 0,4 | <0.01 |
| **minLOT [ms]** | 18,5 ± 2,2 | 18,1 ± 0,1 | 0,05 |
| **maxLOT [ms]** | 442 ± 160 | 364 ± 113 | <0.01 |
| **CLNS_100_ [%]** | 14 ± 10 | 16 ± 5 | 0,13 |
| **CLNS_50_ [%]** | 5,2 ± 3,6 | 7,4 ± 2,8 | <0.01 |
| **CLNS_30_ [%]** | 2,1 ± 1,5 | 3,2 ± 1,3 | <0.01 |
| **CLNS_20_ [%]** | 0,4 ± 0,3 | 0,6 ± 0,3 | <0.01 |
| **CLNS_pt,20_ [%]** | 6,9 ± 3,8 | 45 ± 12 | <0.01 |

*Table S4: Relative LOT statistics for the two subsamples at CFNS_75_> 75^th^ percentile and CFNS_75_ < 25^th^ percentile, respectively, and p-value of the t-Student test.*

|  | **CFNS_75_> 75^th^ prctile** | **CFNS_75_ < 25^th^ prctile** | **p-value** |
| --- | --- | --- | --- |
| **mFLOT [%]** | 0,5 ± 0,1 | 0,7 ± 0,1 | <0.01 |
| **sdFLOT [%]** | 0,22 ± 0,04 | 0,32 ± 0,03 | <0.01 |
| **mdFLOT** | 0,5 ± 0,1 | 0,8 ± 0,1 | <0.01 |
| **moFLOT** | 1,0 ± 0,1 | 0.997 ± 0,003 | 0,15 |
| **MinFLOT** | 0,046 ± 0,013 | 0,053 ± 0,011 | <0.01 |
| **MaxFLOT** | 1,00 ± 0,01 | 0,997 ± 0,003 | 0,63 |
| **CFNS_5_ [%]** | 0,6 ± 0,8 | 0,3 ± 0,8 | 0,03 |
| **CFNS_10_ [%]** | 3,5 ± 2,2 | 4,2 ± 1,9 | <0.01 |
| **CFNS_50_ [%]** | 51 ± 17 | 29 ± 6 | <0.01 |
| **CFNS_75_ [%]** | 86 ± 6 | 44 ± 9 | <0.01 |
| **CFNS_90_ [%]** | 92 ± 4 | 53 ± 11 | <0.01 |

*Table S5: Modulation for the two subsamples at CFNS_75_> 75^th^ percentile and CFNS_75_ < 25^th^ percentile, respectively, and p-value of the t-Student test.*

|  | **CFNS_75_> 75^th^ prctile** | **CFNS_75_ < 25^th^ prctile** | **p-value** |
| --- | --- | --- | --- |
| **nOC** | 0,4 ± 0,1 | 0,4 ± 0,1 | 0,45 |
| **PSTV** | 3,4 ± 1,0 | 5,8 ± 1,8 | <0.01 |
| **EPSTV_1,1_** | 3,4 ± 1,0 | 5,8 ± 1,8 | <0.01 |
| **EPSTV_0,1_** | 1,4 ± 0,5 | 2,5 ± 1,0 | <0.01 |
| **EPSTV_1,0_** | 2,0 ± 0,6 | 3,3 ± 0,9 | <0.01 |
| **LOTV** | 0.95 ± 0.01 | 0,92 ± 0.02 | <0.01 |
| **ELOTV_1_** | 0.05 ± 0.1 | 0.08 ± 0.02 | <0.01 |
| **ELOTV_2_** | 0,08 ± 0.02 | 0 13 ± 0.03 | <0.01 |
| **ELOTV_3_** | 0,11 ± 0,03 | 0,17 ± 0,03 | <0.01 |
| **ELOTV_4_** | 0,13 ± 0,03 | 0,20 ± 0,04 | <0.01 |
| **ELOTV_5_** | 0,15 ± 0,04 | 0,23 ± 0,04 | <0.01 |
| **MI** | 7,4 ± 1,5 | 8,7 ± 2,1 | <0.01 |
| **mSI** | 19 ± 7 | 26 ± 7 | <0.01 |
| **mdSI** | 20 ± 8 | 28 ± 9 | <0.01 |
| **sdSI** | 11 ± 4 | 16 ± 5 | <0.01 |
| **MSA** | 32,5 ± 0,7 | 32,5 ± 0,4 | 0,72 |

*Table S6: Geometry for the two subsamples at CFNS_75_> 75^th^ percentile and CFNS_75_ < 25^th^ percentile, respectively, and p-value of the t-Student test.*

|  | **CFNS_75_> 75^th^ prctile** | **CFNS_75_ < 25^th^ prctile** | **pvalue** |
| --- | --- | --- | --- |
| **L0NS [%]** | 1,6 ± 1,5 | 2,2 ± 2,2 | 0,02 |
| **L1NS [%]** | 22 ± 5 | 25 ± 6 | <0.01 |
| **L2NS [%]** | 76 ± 6 | 72 ± 8 | <0.01 |
| **nCC** | 1,0 ± 0,2 | 1,3 ± 0,4 | <0.01 |
| **lengthCC [leaves]** | 11 ± 3 | 9 ± 3 | <0.01 |
| **TA [leaves]** | 12 ± 5 | 13 ± 4 | 0,02 |
| **fDISC [%]** | 7,9 ± 11,6 | 28 ± 19 | <0.01 |
| **CLS [%]** | 82 ± 7 | 82 ± 5 | 0,45 |
| **CLS_in_ [%]** | 0,5 ± 0,8 | 2,1 ± 1,9 | <0.01 |
| **CLS_in.disc_ [%]** | 4,2 ± 3,6 | 6,5 ± 3,0 | <0.01 |
| **CLS_in.area_ [%]** | 1,8 ± 2,4 | 7,0 ± 5,2 | <0.01 |
| **CLS_in,area,disc_ [%]** | 19 ± 16 | 24 ± 8 | <0.01 |
| **centroid [leaves]** | 5,3 ± 1,8 | 5,3 ± 2,6 | 0,83 |
